# The DecNef collection, fMRI data from closed-loop decoded neurofeedback experiments

**DOI:** 10.1038/s41597-021-00845-7

**Published:** 2021-02-23

**Authors:** Aurelio Cortese, Saori C. Tanaka, Kaoru Amano, Ai Koizumi, Hakwan Lau, Yuka Sasaki, Kazuhisa Shibata, Vincent Taschereau-Dumouchel, Takeo Watanabe, Mitsuo Kawato

**Affiliations:** 1 Computational Neuroscience Labs, ATR Institute International, 619-0288 Kyoto, Japan; 2 Center for Information and Neural Networks (CiNet), National Institute of Information and Communications Technology, 565-0871 Osaka, Japan; 3 Sony Computer Science Laboratories, Inc., 141-0022 Tokyo, Japan; 4 Department of Psychology, UCLA, 90095 Los Angeles, CA USA; 5 Brain Research Institute, UCLA, 90095 Los Angeles, CA USA; 6 Department of Psychology, University of Hong Kong, Pok Fu Lam, Hong Kong; 7 State Key Laboratory for Brain and Cognitive Sciences, University of Hong Kong, Pok Fu Lam, Hong Kong; 8 Department of Cognitive, Linguistic and Psychological Sciences, Brown University, 02912 Providence, RI USA; 9 RIKEN Center for Brain Science, 351-0198 Saitama, Japan; 10 RIKEN Center for Advanced Intelligence Project, 619-0288 Kyoto, Japan

**Keywords:** Operant learning, Reward, Magnetic resonance imaging, Neural decoding

## Abstract

Decoded neurofeedback (DecNef) is a form of closed-loop functional magnetic resonance imaging (fMRI) combined with machine learning approaches, which holds some promises for clinical applications. Yet, currently only a few research groups have had the opportunity to run such experiments; furthermore, there is no existing public dataset for scientists to analyse and investigate some of the factors enabling the manipulation of brain dynamics. We release here the data from published DecNef studies, consisting of 5 separate fMRI datasets, each with multiple sessions recorded per participant. For each participant the data consists of a session that was used in the main experiment to train the machine learning decoder, and several (from 3 to 10) closed-loop fMRI neural reinforcement sessions. The large dataset, currently comprising more than 60 participants, will be useful to the fMRI community at large and to researchers trying to understand the mechanisms underlying non-invasive modulation of brain dynamics. Finally, the data collection size will increase over time as data from newly run DecNef studies will be added.

## Abstract (summary)

**Table:** 

Measurement(s)	functional brain measurement
Technology Type(s)	3 T MRI scanner • functional magnetic resonance imaging • Neurofeedback • machine learning
Factor Type(s)	type of task performed: visual, preference, perceptual, or memory task
Sample Characteristic - Organism	Homo sapiens

Machine-accessible metadata file describing the reported data: 10.6084/m9.figshare.13578008

## Background & Summary

Neurofeedback, based on both functional magnetic resonance imaging (fMRI) and non-fMRI techniques, has seen a dramatic increase in the number of published studies in the last decade^1^. Decoded neurofeedback (DecNef), a form of closed-loop fMRI neurofeedback combined with machine learning approaches, is a more fine-grained rendering of the long-held goal of manipulating brain dynamics or representations^2,3^. As opposed to univariate approaches, where one measures the overall activity level within a region-of-interest (ROI), by treating each voxel in isolation, multivoxel pattern analysis (MVPA)^4,5^ is based on algorithms that learn to decode information distributed in patterns of activity. Because DecNef leverages MVPA, rather than using univariate approaches, it has high target specificity^1,6,7^. Furthermore, while participants know that a neurofeedback experiment is taking place, they are unaware of the content and purpose of the manipulation - reducing confounds due to cognitive processes, or knowledge about the manipulated dimension^7,8^. In its most recent declination, the experimenter can even infer the target neural representation indirectly from surrogate participants through a method called hyperalignment^9,10^. With such a functional alignment approach, patterns of neural activity across participants are used to construct a common, high-dimensional space through a set of linear transformations. These transformations are effectively parameters, and can be used to bring any new data patterns to/from an individual’s brain coordinate system and the model space coordinates^9^.

These aspects make DecNef an attractive tool to develop novel clinical applications, particularly in the context of neuropsychiatric disorders^7,11–15^. Besides clinically-oriented studies, DecNef can be used as an important paradigm in systems and cognitive neuroscience to study basic functions of the brain^10,16^. Several DecNef experiments have been completed to date, targeting representations and cognitive or psychological processes at different levels of the cortical hierarchy: vision and perceptual learning in early visual cortex^3,17^, subjective preference in cingulate cortex^18^, as well as perceptual confidence in a frontoparietal network^19,20^.

**Fig. 1:**
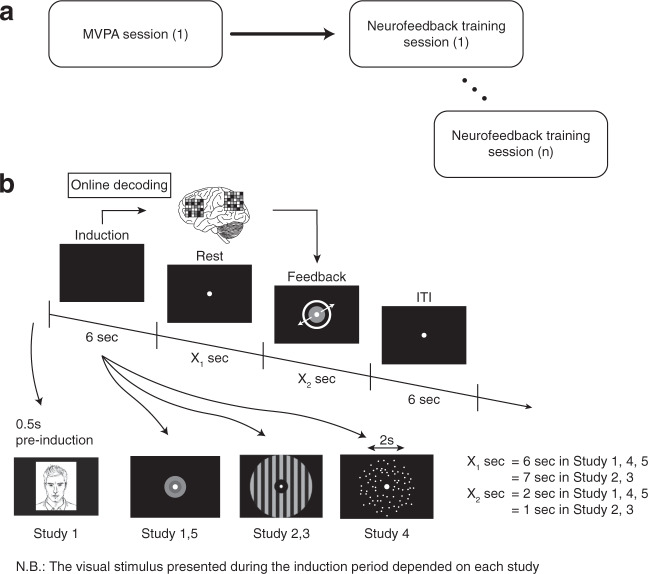
Schematic overview of the experimental design(s). (**a**) Each study had a first fMRI session dedicated to acquire the data necessary to construct a ‘decoder’, a machine learning classifier of brain activity patterns. (**b**) All studies shared the same basic trial design: a first period of 6 seconds called ‘induction’, during which participants were instructed to “modulate, or change their brain activity in order to maximize the feedback received later in the trial”. Unbeknownst to participants, brain activity patterns were fed to the previously trained decoder to calculate the likelihood that the current brain state corresponded to the target state. The likelihood was fed back to participants in the form of a disc within a circle: the larger the disc, the larger the obtained reward. The circle indicated the maximum attainable reward. Beyond this basic structure, the design was slightly modified to meet the requirement of each individual study. In study 1, a neutral face was presented for 0.5 second before the onset of the induction period. Note that in the original study actual face images were used, not cartoons. In study 2 and 3, instead of a simple visual cue indicating the induction period, participants were presented with an achromatic grating. Lastly, in study 4, a random dot kinetogram with 0% coherence was shown for 2 seconds at variable onsets during the induction period.

Briefly, the goal of each study contained in this data collection was to target and non-invasively modulate a specific representation in the brain. Notwithstanding that each experiment probed a different cognitive process or mental representation, all studies used the same basic design logic (Fig. 1a): (1) an initial session to acquire fMRI data that was used to train the machine learning algorithm - the MVPA or decoder construction session; 2) subsequent neurofeedback sessions, ranging from 2 to 5 days, depending on the study (described in detail in the “Data Records” section). In the decoder construction session, participants performed a simple visual (Study 2, 3), preference (Study 1), perceptual (Study 4), or memory task (Study 5), depending on the study target, while in the neurofeedback sessions the procedure was nearly identical in all cases (Fig. 1b). During neurofeedback training, participants were instructed to modulate or manipulate their brain activity in order to maximize the size of a feedback disc shown on the screen towards the end of each trial. The size of the disc reflected the amount of reward obtained on that single trial (maximum JP¥300), which was then summed to a terminal reward. Participants were told that the goal of the task was to maximize the amount of reward (bonus of up to JP¥3000, on top of JP¥8000). Unbeknownst to them, what really determined the size of the feedback disc - and therefore the amount of reward - was the likelihood that their current brain state corresponded to a target state. That is, the previously trained decoder was used in real time to infer how likely it was that a target activity pattern had occurred.

As of yet, only a few research groups worldwide have had the opportunity to run such technically challenging experiments. We have published elsewhere a description on how to run decoded neurofeedback experiments^10^. Nevertheless, a key aspect of DecNef (and neurofeedback in general) remains rather elusive: what is the underlying neural mechanism? Recent work has begun to explore this question through experiments, meta-analyses, computational models, and neural network simulations^6,21–26^. Reinforcement learning has been suggested as one plausible mechanism^6,22^, but the field would considerably benefit from having access to a rich dataset, comprising more than 60 individuals and totalling more than 200 hours of fMRI scanning time under DecNef training. This data collection consists of 5 independent studies that shared the same overall experimental design. Table 1 summarises all studies, indicating the relevant publication(s), and the main target of the neurofeedback process training.

**Table 1:** Summary of the studies incorporated in the data collection.

	Author, Journal, Publication Year	Target process	Target brain area
Study 1	Shibata *et al. PLoS Biol* (2016)	Face preference	Cingulate cortex
Study 2	Amano *et al. Curr Biol* (2016)	Colour-orientation association	Early visual cortex
Study 3	Koizumi *et al. Nat Hum Behav* (2016)	Conditioned fear reduction	Early visual cortex
Study 4	Cortese *et al. Nat Commun* (2016), *NeuroImage* (2017)	Perceptual confidence	Inferior parietal and dorsolateral prefrontal cortex
Study 5	Taschereau-Dumouchel *PNAS* (2018), *Molecular Psychiatry* (2019)	Common fear reduction	Ventral temporal cortex

Finally, it is important to highlight the future growth potential of the DecNef data collection. Besides data generated through our own collaborative efforts, we have recently released software to perform DecNef experiments^10,27^, under agreement terms that all data shall be made available to the wider scientific community through the DecNef data collection. As such, this cross-country scientific effort will result in new datasets being added semi-automatically to this data sharing project.

## Methods

### Ethics statement

All participants in all studies provided written informed consent. All recruitment procedures and experimental protocols were approved by the institutional review board at the Advanced Telecommunications Research Institute International (ATR, Kyoto, Japan), (approval numbers: 14–121, 12–120, 15–181, 14–140, and 16–181), and conducted in accordance with the Declaration of Helsinki.

### Participants

Table 2 summarizes the demographic characteristics of participants included in each study.

**Table 2:** Participants demographics.

	Sample size	Female/Male	Age (y) [mean ± std]
Study 1	24	4/20	22.3 ± 2.2
Study 2	12	1/11	21.2 ± 1.8
Study 3	9 (17)	3/6 (6/11)	23.6 ± 3.6 (23.5 ± 2.8)
Study 4	10	3/7	24.3 ± 3.1
Study 5	12 (17)	4/8 (6/11)	22.5 ± 2.7 (22.6 ± 2.3)

For Study 3 and 5, consent for data sharing was obtained only from a subgroup of participants. The demographics are reported for both the subgroup and the original group, the latter in brackets.

### Source data

Technical details about scanning parameters used in all studies are given below, and those that differed between studies, in Table 3.

**Table 3:** Scanning parameters that differed between studies.

	Scanner type (all 3 T)	Echo time (TE)	Head coils (channels)
Study 1	Siemens MAGNETOM Verio	26 ms	12
Study 2	Siemens MAGNETOM Verio	26 ms	12
Study 3	Siemens MAGNETOM Trio	30 ms	12
Study 4	Siemens MAGNETOM Trio	26 ms	12
Study 5	Siemens MAGNETOM Verio, Prisma	30 ms	12 (Verio), 20 (Prisma)

Participants were scanned in 3 T MRI scanners (see Table 3 for details) with a head coil (see Table 3 for details, e.g., number of channels) at the ATR Brain Activation Imaging Center. In all experiments, 33 contiguous slices were acquired (TR = 2000 ms, voxel size = 3 × 3 × 3.5 mm^3^, field-of-view = 192 × 192 mm, matrix size = 64 × 64, slice thickness = 3.5 mm, 0 mm slice gap, flip angle = 80 deg) with interleaved order (e.g., [1 3 5…, 2 4 6…]), oriented parallel to the AC-PC plane, covering the entire brain. T1-weighted MR images were also obtained (magnetization-prepared rapid gradient-echo or MP-RAGE; 256 slices, TR = 2250 ms, TE = 3.06 ms, voxel size = 1 × 1 × 1 mm^3^, field-of-view = 256 × 256 mm, matrix size = 256 × 256, slice thickness = 1 mm, 0 mm slice gap, TI = 900 ms, flip angle = 9 deg).

All data were converted from raw DICOM images to anonymized Nifti files, the standard format in neuroimaging (Neuroimaging Informatics Technology Initiative), with the software *dcm2niix*^28^. The Nifti files from this data collection are provided as such.

## Data Records

The data collection can be accessed from our institutional repository “DecNef Project Brain Data Repository” (https://bicr-resource.atr.jp/drmd/), or from the Synapse data repository (10.7303/syn23530650)^29^. See “Usage Notes” for a detailed explanation on how to get access to, and download the data.

**Fig. 2:**
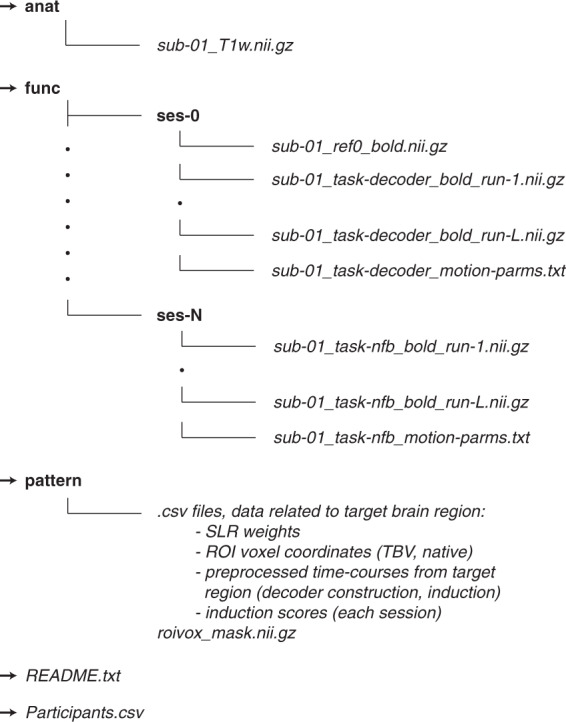
Dataset structure and content. The structure is for a representative participant, and is identical for all participants. Words in **bold** correspond to folders, while words in *italic* relate to files. Please note that the number of participants, days (1-N), and runs (1-L) vary study-by-study.

The data is organized according to the structure illustrated in Fig. 2. Briefly, for each study, the top folder contains a folder for each participant (e.g., “sub-01”). Within it there are three subfolders, “anat” with the raw Nifti files related to structural/anatomy scans, “func” - which is further subdivided into session-specific folders (e.g., “ses-0”: decoder, “ses-1”: first session of neurofeedback, etc.) containing all compressed Nifti files from the functional scans. Each of these subfolders additionally include the reference functional image used for re-alignment and the motion parameters computed offline. The third folder “pattern” contains preprocessed data related to the targeted brain region(s) from the decoder construction session and from the neurofeedback sessions. Preprocessed data include trial-by-trial multivoxel activity patterns corresponding to the voxels selected by the decoder algorithm (for both decoder and neurofeedback sessions)); the weights assigned to each voxel by the decoder algorithm and used in the real-time classification to infer the likelihood; the induction performance for neurofeedback sessions (i.e., the trial-by-trial likelihood of decoding the target activity pattern). Finally, the folder also contains the coordinates in native and native-TBV space of the voxels used by the decoder to compute the neurofeedback scores. The file “*README.txt*”, included in each study folder, provides details on the data content. The file “*Participants.csv*” provides demographics information on participants, as well as, where relevant, information on the training group to which participants were assigned (e.g., Study 1: increase or decrease of preference to neutral faces). The number of participants, number of neurofeedback sessions, and the target of training are summarised in Table 4.

**Table 4:** Number of participants and neurofeedback training sessions, along with the targeted process and brain area.

	# participants	# neurofeedback sessions	Target of training
Study 1	24	3	face preference in cingulate cortex
Study 2	12	3	red colour in early visual cortex
Study 3	9 (17)	3	red/green colour in early visual cortex
Study 4	10	4 (2 x training direction)	high/low confidence in inferior parietal and dorsolateral prefrontal cortex
Study 5	12 (17)	5	specific animal representation in ventral temporal cortex

For Study 3 and 5, consent for data sharing was acquired only from a subgroup of participants. We report the number of participants for both the subgroup and the original group, the latter in brackets.

## Technical Validation

### Data anonymization procedure

**Fig. 3:**
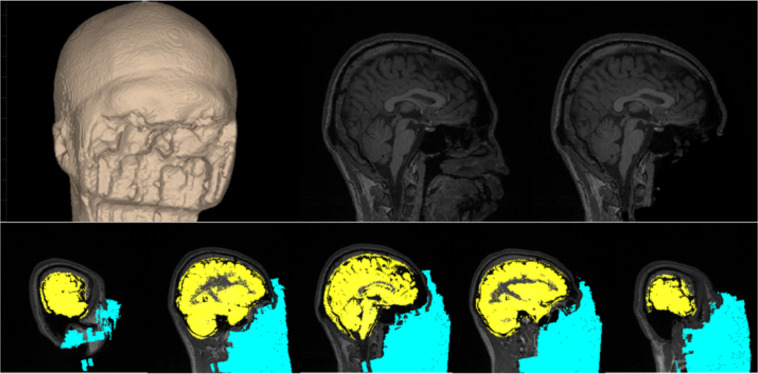
Anonymization (defacing) of an example participant’s structural scan.

High resolution anatomical scans were defaced to ensure proper anonymization of structural data. Images were bias-corrected with the Statistical Parametric Mapping (SPM) Toolbox (https://www.fil.ion.ucl.ac.uk/spm/) version 12, and defacing was performed with the automated defacing tools from the FreeSurfer suite^30^ (see Fig. 3 for the resulting image in an example participant).

### Real-time correction of head motion

Given the fine-grained, high spatial resolution requirements of the DecNef approach, a crucial aspect of a successful study pertains to a technical detail. The functional alignment of brain images used for online feedback computations need to have a very high between-sessions consistency. That is, the images have to be aligned to the original decoder construction and the alignment has to be precise at the (sub)voxel level. Even small head movements easily break this prerequisite, leading to imperfect decoding and feedback score calculations. To ensure this requirement, all studies monitored in real time the alignment between the incoming functional brain images and a template from the original decoder construction. Although Turbo BrainVoyager (TBV, Brain Innovation) was used to correct for head motion in real-time - and the displacement could be viewed by the experimenter throughout, there is no guarantee that the corrected images will be meaningful in terms of decoding, especially if there was a sudden, marked displacement.

Therefore, during real-time neurofeedback experiments, the following processing steps were applied to the raw DICOM images. First, measured functional images during the induction period underwent three-dimensional motion correction using TBV. Second, time-courses of signal intensities were extracted from each of the voxels identified by the decoder, and were shifted by 6 seconds to account for the hemodynamic delay. Third, a linear trend was removed from the time course, and the signal time course was z-score normalized for each voxel using signal intensities measured from 10 s after the onset of each fMRI run. Fourth, the data samples to calculate the feedback score were created by averaging the BOLD signal intensities of each voxel over the induction period (i.e., 3 TRs).

### Functional alignment of multivoxel patterns between decoder construction and DecNef sessions

**Fig. 4:**
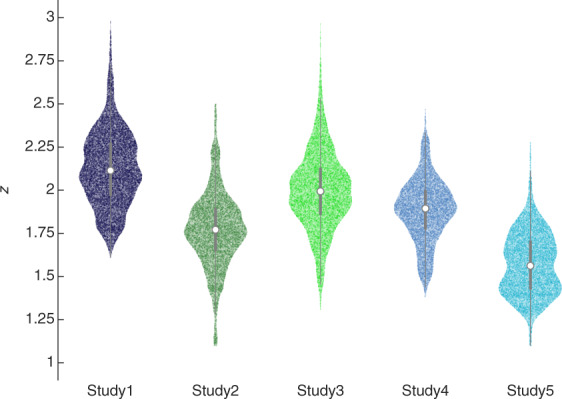
Fisher transformed correlation between mean activity patterns and real-time activity patterns. High values indicate better functional alignment between the real-time measured pattern and decoder construction pattern. Each dot represents the correlation value for one trial. For each violin plot there are N (days) × M (participants) × L (runs) × J (trials) dots. The white circle at the centre of the plots represents the median, the thick grey line, the interquartile range, and the thin grey line the adjacent values. Data points with *z* < 1.10 were removed from the plot.

An efficient way to control for data quality in terms of the target voxels’ activation pattern is to first compute their mean activation (with data from the initial decoder construction session); then, in the real-time sessions, calculate the trial-by-trial correlation between the mean pattern and the incoming activity pattern. This approach ensures that upon significant changes in the voxels’ response patterns due to, e.g., head or body motion, a decrease of the correlation would rapidly occur and thus can be detected. At optimal levels the correlation should be within the interval r ∈[0.85 1.00], or Fisher transformed z ∈[1.26 Inf]. We confirmed that this was the case in all studies (Fig. 4), with, on average, less than 2% of the trials having z < 1.26 (Study 1: 0.13%, Study 2: 3.17%, Study 3: 0.91%, Study 4: 0.36%, Study 5: 3.74%).

### Pattern correlation measure and head motion

**Fig. 5:**
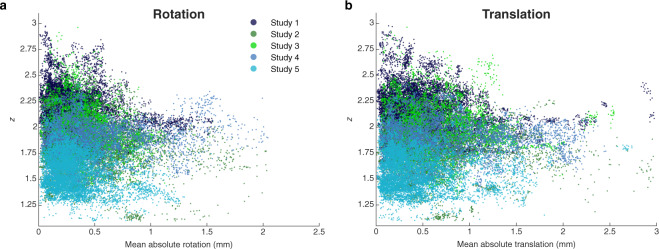
Pattern correlation vs head motion. Head motion was computed as rotation in the 3D directions (**a**) or as translation in the 3D directions (**b**), and plotted against Fisher transformed pattern correlation coefficients. Data points from different studies are plotted with different colours. Each data point represents a trial from a given participant, run, and session. Statistical significance was assessed with linear mixed effects models (Tables 5, 6).

We next established the relationship between head motion and the pattern correlation measure. Head motion parameters were computed with SPM12, yielding 3 parameters for translation and 3 parameters for rotation. For the purpose of this analysis, we computed the mean absolute rotation and mean absolute translation by averaging the relevant 3 parameters, over the 3 TRs used in the neurofeedback experiment to compute the decoder likelihood and the pattern correlation. These two indexes of head motion (in mm) are plotted against the Fisher transformed correlation coefficients (i.e., the pattern correlation measure) pooled across all studies (Fig. 5). For statistical analysis, the single trial data were concatenated and analysed with linear mixed effects (LME) models (in Wilkinson notation, specified as *y* ~ *1* + *m* + (*1* | *st*) + (*1* + *st | prt)*; where *y*: pattern correlation, *1*: intercept, *m*: motion parameter, *st*: study, *prt:* participant). Specifically, these LME models were designed such that motion was treated as a fixed effect, study as a random effect and covariate, and individual participants as random effects nested within studies. Each model was fitted on the corresponding motion parameter (see Tables 5 and 6, respectively). The significant couplings with parameter values ≪0 for the fixed effect ‘motion’ indicate that pattern correlation was negatively influenced by head motion. Interestingly, pattern correlation appeared more sensitive to rotation movements.

**Table 5:** LME model to analyse the effect of motion (rotation) on pattern correlation.

y = pattern correlation					
formula (Wilkinson notation)	*y ~ 1* + *m* + (*1 | s*t) + (*1* + *st | prt)* *where m: motion reg.: rotation, st: study, prt: participants*				
Fixed effect	β (SE)	tStat	DF	*P*	*CI*
(Intercept)	0.614 (0.048)	12.91	36634	4.72 × 10^−38^	[0.520 0.707]
Mean rotation	−0.107 (0.007)	−15.21	36634	4.44 × 10^−52^	[−0.121 −0.093]
**Random effects**	**Name-1**	**Name-2**		**Type**	**Estimate**
study (5)	intercept	intercept		std	0.060
participants (67)	intercept	intercept		std	0.610
	study	intercept		corr	−0.940
	study	study		std	0.139
error	sqrt (dispersion)				0.200

**Table 6:** LME model to analyse the effect of motion (translation) on pattern correlation.

y = pattern correlation					
formula (Wilkinson notation)		*y ~ 1* + *m* + *(1 | st)* + *(1* + *st | prt)* *where m: motion reg.: translation, st: study, prt: participants*			
Fixed effect	β (SE)	tStat	DF	*P*	*CI*
(Intercept)	0.926 (0.065)	14.14	36709	2.82 × 10^−45^	[0.797 1.054]
Mean translation	−0.169 (0.012)	−13.56	36709	8.94 × 10^−42^	[−0.194 −0.145]
**Random effects**	**Name-1**	**Name-2**		**Type**	**Estimate**
study (5)	intercept	intercept		std	0.089
participants (67)	intercept	intercept		std	0.761
	study	intercept		corr	−0.939
	study	study		std	0.173
error	sqrt (dispersion)				0.355

### Additional sources of noise

Finally, other sources of physiological noise (e.g., heartbeat, or respiration) will also impact the multivoxel activity patterns used for real-time decoding. These sources were not directly measured in the present studies, and therefore we may only speculate on their influence. If their effects were relatively homogenous across all voxels, we expect little to no impact on the pattern correlation measure because the relationship between voxels would remain essentially unchanged. In the presence of uneven effects across voxels, we would see the pattern correlation affected similarly as with head motion. One may worry that in the first case we would not be able to detect noise distortions of the data, thus potentially invalidating the veracity of the target likelihood fed back to participants. But because all voxels’ activities first underwent baseline normalization, and then the feedback likelihood was calculated as the dot product between the voxels’ activity pattern and a weight vector, what matters is the pattern of voxels (the ‘difference’ between voxels activities). It should be noted, therefore, that due to the specificity of MVPA, it is unlikely that additional sources of noise would have significantly affected the information content of voxels’ activity patterns without also affecting the pattern correlation measure.

## Usage Notes

The data is available for the purposes of scientific research, technology development, and education under the auspices of an academic, research, government or commercial entity, and is stored at https://bicr-resource.atr.jp/drmd/. Applicants should first read the privacy statement of the data collection, fill the registration form and submit their application through the website linked above. Upon clearance, each dataset will be downloadable separately. The DecNefPro consortium (https://bicr.atr.jp/decnefpro/), where the data collection is hosted, is formally recognized as a national database by the Japan Agency for Medical Research and Development (AMED), an official Agency of the Japanese Government.

Additionally, the data collection can also be accessed under the same terms at Synapse (10.7303/syn23530650).

Users of the DecNef data collection are encouraged to formally cite this publication.

## Data Availability

To convert DICOM images to Nifti files, the open sources software *dcm2niix* is freely available at https://github.com/rordenlab/dcm2niix. To construct machine learning decoders, all studies employed the freely available Sparse Logistic Regression Toolbox, implemented in Matlab. The toolbox implements classification with sparsity prior and is available for both binary and multiclass problems. The toolbox can be found at https://bicr.atr.jp/~oyamashi/SLR_WEB.html. Software to run DecNef experiments can be obtained freely upon reception of a signed agreement form, and participating the resulting data towards the DecNef data collection. The usage of the software is intended for academic purposes. The software is implemented in Matlab and can be found at https://bicr.atr.jp/decnefpro/software/.
